# Perceived effectiveness of pictorial health warnings on changes in smoking behaviour in Asia: a literature review

**DOI:** 10.1186/s12889-018-6072-7

**Published:** 2018-10-11

**Authors:** Suci Puspita Ratih, Dewi Susanna

**Affiliations:** 1 Faculty of Public Health, Universitas Indonesia, Depok, 16424 West Java Indonesia; 2 Department of Public Health, Faculty of Sports Sciences, Universitas Negeri Malang, Malang, 65145 East Java Indonesia; 3 Department of Environmental Health, Faculty of Public Health, Universitas Indonesia, Depok, West Java 16424 Indonesia

**Keywords:** Tobacco, Tobacco control, Graphic warning, Cigarette packs

## Abstract

**Background:**

Several Asian countries have implemented pictorial health warnings on cigarette packs as suggested by the World Health Organization with various policies based on countries’ systems. The study is aimed to analyse multiple research studies on the perceived effectiveness of Pictorial Health Warnings as a deterrent to smoking intention and as a stimulant of smoking behaviour in smokers and non-smokers in Asian countries.

**Method:**

Peer-reviewed articles were identified through multiple science databases indexed by Scopus, MEDLINE or PubMed. The review was limited to articles that reported original research findings, were conducted in Asian countries and were available for review by January 2010. A PRISMA Flow diagram was used to identify the articles through the process of data screening and extractions.

**Result:**

A total of 14 original articles which met the criteria were included in the review, consisting of 12 quantitative studies and 2 studies with both quantitative and qualitative methods from 17 jurisdictions. The reviewed studies found that pictorial health warnings were associated with a greater perception of salience (reading and noticing the warning), emotional effects, and cognitive increase. Additionally, in the reviewed studies, pictorial health warnings were perceived as more effective in deterring smoking initiation and encouraging smoking cessation than text-only warnings. Several studies also evaluated the effectiveness of a new pictorial warning compared with the old one. However, the relevance of refreshing the pictures of pictorial health warnings and the length of the effective period of the implementation of pictorial health warnings were less frequently studied.

**Conclusion:**

Pictorial health warnings perceived as more effective in deterring smoking initiation among non-smokers and as well as in stimulating smoking cessation among smokers. Future studies on pictorial health warnings should study the relevance of changing or refreshing pictorial health warning on cigarette packages in a period of time.

## Background

Asia, with its varied political systems and vast population, has the highest number of tobacco consumers and is the main target of transnational tobacco industries, especially China, India, and Indonesia [1]. To address tobacco issue in Asia, the World Health Organization (WHO) has assisted the member states in this continent to implement WHO’s Framework Convention on Tobacco Control (FCTC) which came into force in 2005. FCTC is global standards and guidelines for tobacco policy among WHO members which successfully improved tobacco control policy in 120 out of 174 countries by 2011 [2]. The WHO’s FCTC is the first global tobacco-control treaty that encourages ratifying countries to develop and implement tobacco control policies in their own countries, regulating about tobacco advertising, tobacco tax and price, smoke-free area, and health warning message on tobacco packages [3].

Graphic health warnings, which make the potential of smoking to cause diseases more real to smokers, both can improve public knowledge, and work to encourage cessation through the generation of concern stimulated by the emotionally charged messages [4]. The use of pictorial warnings is of special relevance in countries with low literacy rate [5]. A study conducted by Scollo and Winstanley [6] found that smokers who smoked 20 cigarettes per day would be exposed to the health warnings around 7000 times each year.

Studies in the USA found that recognition of warnings in pictorial health warnings was higher than those in text-only warning. Additionally, the lung cancer warning discouraged adolescent non-smokers from wanting to smoke [7, 8]. Pictorial warnings have been found to be effective in deterring smoking, especially among the young [9]. However, Li, Chan, and Lam [10], who conducted a study on smoking behaviour among Hong Kong Chinese women, found that current, ex- and never smokers thought that smoking cessation advertisements were less strong than anti-drug advertisements. Nevertheless, current and ex-smokers in the study were aware of pictorial health warnings on cigarette packs which showed varying degrees of horror and disgust.

However, evidence found the large health warnings as it has been documented that larger health warnings covering more of the front of the pack are more effective than smaller warnings [11]. It is also supported by psychological theories which argued the better recall of larger warning [12]. Moreover, refreshing the pictures on pictorial health warnings regularly in a period of time was known to help to sustain the effects on smokers [13]. Thus, this study is aimed to analyse multiple research studies conducted in Asian countries to describe the perceived effectiveness of Pictorial Health Warning (PHWs) in deterring smoking initiation among non-smokers and stimulating smoking cessation among smokers.

## Methods

### Data sources

Peer-reviewed articles were identified through multiple science databases: Sciencedirect, ProQuest, Oxford Journals, SpryngerLink, SAGE and Scopus which are indexed to Scopus, Medline or PubMed. We also included Google Scholar as the source of scientific papers to search for more articles that might not be published in the indexed journals. Electronic searches were conducted to identify relevant literature. The following keywords were used to identify relevant articles: (“pictorial warning”; “graphic warning”; “health warning”) with at least one of the following terms: smoking, tobacco, cigarette, product, package, and pack. All articles appearing in the search results were listed.

The review was limited to articles that reported original research findings and were published from January 2007 to Jathe nuary 2017. Studies which do not content aspects of warning, packaging and labelling regulation were excluded, as well as studies conducted in other Asian countries. Due to the diversity of research methods in this domain, the reviewer did not restrict studies to a particular design; however, each of the articles were reviewed for the following methodological criteria: (1) objectives and/or research questions were clearly explained, (2) sample and/or study population are described, (3) data collection method is consistent, (4) key measurements are adequate and valid, (5) results are clearly defined and measured (6) analysis of findings are clear and appropriate.

### Data extraction

In conducting the data extraction, we firstly excluded articles that did not include Asian settings and/or did not sufficiently represent Asian countries as their study locations. A total of 141 articles were identified by titles which resulted in 14 articles excluded due to duplications. The 127 articles presenting empirical data were identified by titles and abstracts, 87 were excluded due to poor aspects of warning, packaging, and labelling. The 40 articles were identified by abstracts and methods which resulted in excluding 26 more articles due to insufficient methodological information. The 14 original articles included in the review consisted of 12 quantitative studies and 2 studies with both quantitative and qualitative components. The strategy of data extraction and identification used a PRISMA flow diagram [14].

## Results

**Fig. 1:**
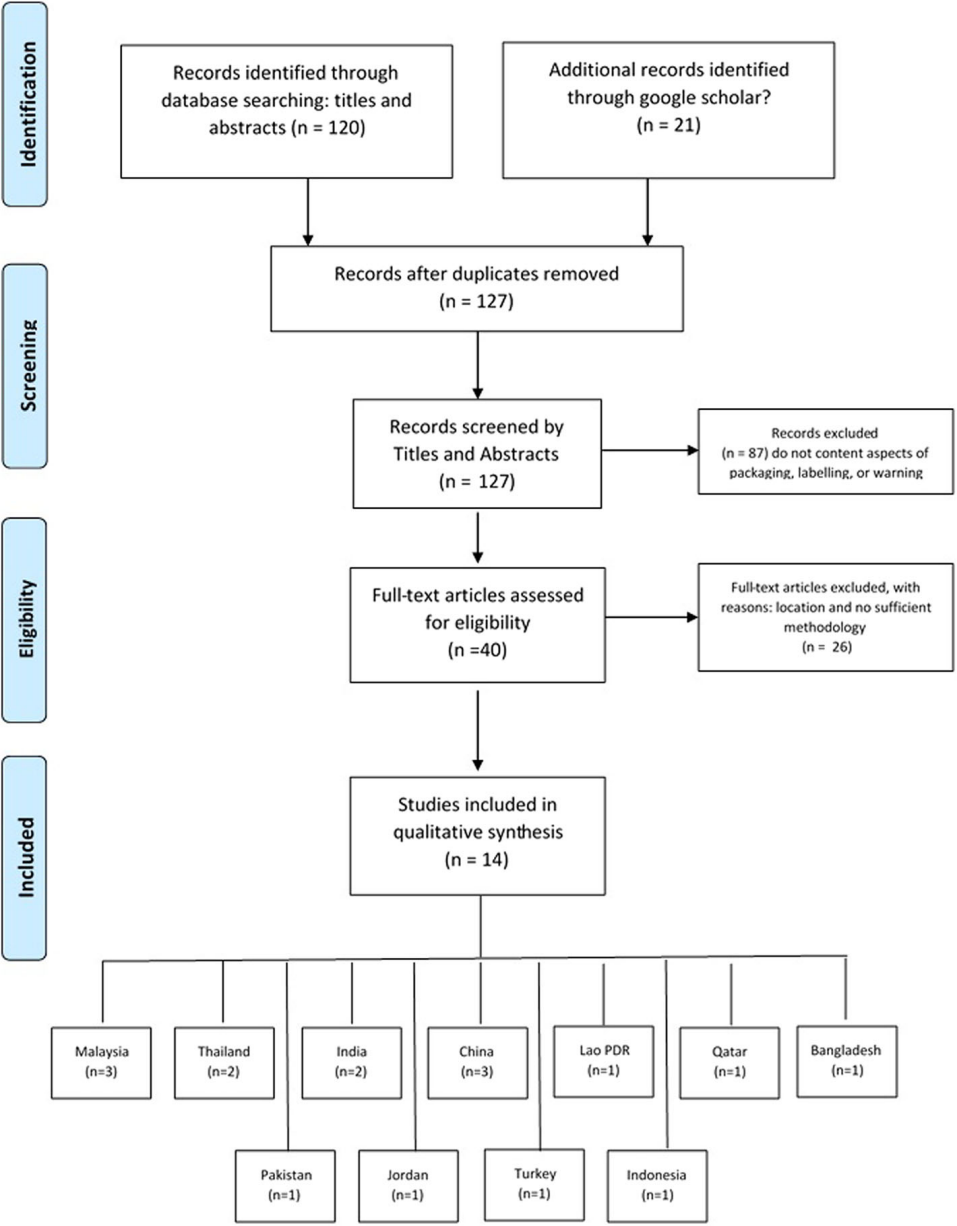
The PRISMA Flow Diagram of The Systematic Search and Data Extractions

After removing duplicates and ineligible articles, 14 studies met the inclusion criteria based on study locations, appropriate variables measured and sufficient study methods. A PRISMA diagram flow can be seen in Fig. 1. The most common reasons for exclusion of candidate articles were because no effect of pictorial warnings on smoking behaviour evaluated, or country of study. A summary of each study is available in online supplementary Table 1. Research articles came from the following jurisdictions: Malaysia (*n* = 3), Thailand (*n* = 2), India (*n* = 2), China (*n* = 3), Lao PDR (*n* = 1), Qatar (*n* = 1), Bangladesh (*n* = 1), Pakistan (*n* = 1), Jordan (*n* = 1), Turkey (*n* = 1), Indonesia (*n* = 1). Several articles included data collected in multiple countries. However, these articles were counted as a single study but recorded in multiple jurisdictions.

**Table 1:** Online Supplementary of Reviewed Studies

Author	Year Of Publication	Country Of Study	Journal	Index	Publisher
Fathelrahman et al.	2010	Malaysia	International Journal of Environmental Research and Public Health	MEDLINE and PubMed	NCBI
Fong et al.	2010	China	Tobacco Control	Scopus	NCBI
Zaidi et al.	2011	Pakistan	BMC Public Health	Scopus	NCBI
Hawari et al.	2011	Jordan	BMC Public Health	Scopus, MEDLINE, PubMED	NCBI
Yong et al.	2013	Thailand and Malaysia	Nicotine & Tobacco Research	Scopus	Oxford Journals
Behera et al.	2013	India	Indian Journal of Health and Wellbeing	EBSCO and ProQuest	Indian Association of Health, Research and Welfare
Tugrul Tugba Orten	2013	Turkey	Asia Pacific Journal of Public Health	MEDLINE and PubMed	SAGE Publications
Awaisu et al.	2013	Qatar	Nicotine & Tobacco Research	Scopus	Oxford Journals
Wu et al.	2014	China	Health Education Journal	Scopus	SAGE Publications
Sychareun et al.	2015	Lao PDR	BMC Public Health	Scopus, MEDLINE, PubMED	NCBI
Elton-Marshall et al.	2015	China and Malaysia	Tobacco Control	Scopus	BMJ Publishing Group
Auemaneekul et al.	2015	Thailand	Asia Pacific Journal of Public Health	MEDLINE and PubMed	SAGE Publications
Dien et al.^a^	2015	Indonesia	–	–	Center for Health Research, Universitas Indonesia
Mutti et al.	2016	India and Bangladesh	Tobacco Control	Scopus	BMJ Publishing Group

^a^The study was published in the ResearchGate site

### Online supplementary and Operationalisations of the studies

No study met the criteria for inclusion prior to 2010. Meanwhile, the most recent study that was included in this study was published in 2016. During that period, the number of studies measuring the effectiveness of pictorial health warnings in Asian countries seems to fluctuate. There are two studies published in 2010, two studies published in 2011, four studies published in 2013, one study published in 2014, four studies published in 2015, and one study published in 2016. Most studies were published in credible international journals indexed by Scopus, MEDLINE, PubMed, ProQuest, and EBSCO. There was only one study published in the ResearchGate site. The online supplementary of the reviewed studies can be seen in Table 1.

Twelve studies used a quantitative method based upon the designs of cross-sectional (*n* = 5), cohort (*n* = 2), survey (*n* = 2), case-control (*n* = 1), or experiment (*n* = 2). The rest of the studies (*n* = 2) used a mix between quantitative and qualitative methods by conducting both a cross-sectional study and a focus group discussion. Regarding the number of the study population, the smallest sample was obtained by Wu et al [15]. who interviewed 202 people derived from a multistage-random sampling. Additionally, in Pakistan, the effectiveness of graphic health warning was measured among 388 high school students [16]. Meanwhile, the study in LAO PDR evaluated the implementation of pictorial health warnings both in average citizens and policy makers [17]. On the other hand, the study by Elton-Marhsall et al. [18] examined a big study population with 6513 adult smokers in China and 2883 adult smokers in Malaysia. Additionally, the study by Mutti et al. [19] had the highest response rate by 98.94% among 2061 respondents. Moreover, all studies performed univariate and bivariate analyses. However, only five studies were found to conduct a multivariate analysis. The operationalisation and statistical analysis of the reviewed studies can be seen in Table 2.

**Table 2:** Operationalisation and Statistical Analysis of Reviewed Articles

Author	Country	Study Period	Study Mehod	Study Design	Data Collection Method	Respondent Selection Method	Respondent	Response/retention Rate	Statistical Analysis
Fathelrahman et al.	Malaysia	May to December 2008	Quantitative	A two-group randomized design (control and intervention groups)	Self-administered questionnaire	Population based	140 male Malaysian Adult	80%	Independent sampe t-test, Chi-square or McNemar statistics, multiple logistic and linear regressions with *p* < 0.05
Fong et al.	China	January to February 2009	Quantitative	A city X sex (two categories) X participant group (adult smokers, adult non-smokers, youth)	Self-administered questionnaire	Population based	1169 adult and youth	–	Chi-square test, a mixed-model and post hoc contrasts
Zaidi et al.	Pakistan	January to February 2010	Quantitative	Experimental	Self-administered questionnaire	Selected Schools	388 high school students	–	Friedman test, Wilcoxon Signed Ranks Test, and Mann Whitney U test with p < 0.05
Hawari et al.	Jordan		Quantitative	Cross-sectional Survey	Self-administered questionnaire	Population based	450 students	79.78%	Chi-square test
Yong et al.	Thailand	Wave 1: Jan-March 2005; Wave 2: July-Sept 2006; wave 3: Jan-march 2008	Quantitative	Cohort survey	Face-to-face interview	a stratified multistage sampling design	3067	78% and 83%	Logistic regression
	Malaysia	Wave 1: Jan-March 2005; wave 2: Agst 2006-March 2007; wave 3: March-Sept 2008					3220	44% and 59%	
Behera et al.	India	August - Oct 2012	Quantitative	Cross-sectional	Face-to-face interview	Selected hospital	308	–	Chi-square test
Tugrul, Tugba Orten	Turkey	2010	Quantitative	Survey			360 undergraduate students	95%	Regression
Awaisu et al.	Qatar	Nov 2011-Jan 2012	Quantitative	Cross-sectional	Face-to-face interview	Selected public places	500	–	chi-square or Fisher’s exact
Wu et al.	China		Quantitative	Cross-Sectional	Face-to-face interview	Multi-stage sampling design	202	–	Chi-square tests
Sychareun et al.	Lao PDR		Quantitative and Qualitative	Cross-Sectional and Indepth Interview	Self-administered questionnaire and interview	Popolation based and Purposive sampling	1360 participants and 15 policy makers	–	chi-square or Fisher’s exact, multiple logistic regression
Elton-Marshall et al.	China	Wave 1: April- Agst 2006; Wave 2: Oct 2007 to Jan 2008; Wave 3: May to October 2009	Quantitative	Survey	Interview	Multistage cluster sampling at wave 1	6513 adult smokers	81.6% and 80.4%	Generalised Estimating Equations (GEE) with 95% CI
	Malaysia	Wave 3: Feb-Sept 2008; wave 4: July-Nov 2009	Quantitative	Cohort survey	Thelephone and face-to-face interveiws	a stratified multistage sampling design	2883 adult smokers	61%	
Auemaneekul et al.	Thailand	July to August 2012	Quantitative and Qualitative	Cross-sectional and FGD	Self-administered questionnaire	multistage stratified random sampling	1239	–	Content analysis, descriptive and odds ratios
Dien et al.	Indonesia	June 2014	Quantitative	Survey	Face-to-face interview	Random sampling	1177	–	t-tests
Mutti et al.	India	10 April-6 August 2012	Quantitative	Experimental (pre-post test)	Face-to-face interview	Population based	1060 adults and 1001 youths	98.94%	Chi-square tests (categorical variables), one-way analysis of variance and t-tests (continuous variables)
	Bangladesh	9 May-18 June 2012							

Empty cells indicate the component was not identified

### Demographic characteristics and smoking behaviour in the studies

The studies obtained data from a variety of demographic characteristics of respondents, including age, sex, occupation, income, level of educations, ethnicity, and nationality. Three studies were conducted among adults (aged more than 18 years old), one study was carried out among teenagers (ranged from 15 to 18 years old), three studies were conducted among youths and young adults (ranged from 15 to 26 years old) and the rest five studies were carried out among both youths and adults (aged < 18 years old and ≥ 18 years old). Twelve studies obtained the data from both male and female respondents, while only one study conducted among male respondents only and the rest did not mention any specific gender characteristic.

Regarding study locations, three studies obtain the data in urban and rural areas; one study obtained the data in urban, semi-urban and rural areas, six studies gathered the data in urban areas only, while the rest of the studies did not specify it. Three studies included ethnic groups in their respondents’ characteristics which were classified as han-chinese and non-han-chinese groups in China and the majority and minority in Thailand and Malaysia. Another study in Qatar also classified their respondents based on their nationality which was divided as Qatari and non-qatari [20]. One study also obtained information about respondents’ medical conditions. Additionally, two studies in Qatar and China also collected information about respondents’ marital status [18, 20]. Additionally, most of the studies classified their respondents based on their education levels which mostly were divided into four groups: illiterate, low (up to middle school), high (up to high school), and graduate (diploma or higher). Five studies obtained information about respondent’s occupations. Moreover, eight studies classified respondents’ incomes which were mostly divided into three groups: low, moderate and high.

### Smoking behaviour

The data in the studies reviewed were obtained from respondents with different smoking behaviours. Eight studies examine both smokers and non-smokers, while six studies examine smokers only. Adult smokers were classified as follow: non-daily smokers, daily smokers, weekly smokers, smokeless tobacco users, and mixed between smokeless and smoked tobacco users. While youth smokers were classified as follow: daily smokers, occasional smokers, smokeless tobacco users, and mixed users. On the other hand, non-smokers were mostly classified into never-smoker, former smoker, susceptible and non-susceptible (for youth). The majority of the studies found that the percentages of male smokers were higher than that of smoking women. It is relatively a result of the higher number of male respondents than female respondents in the studies.

Compared with China and Thailand, smokers in Malaysia smoked fewer cigarettes with the average of 0–10 cigarettes per day [13, 18]. However, another study found that the majority of smokers in Malaysia smoked 11–20 cigarettes per day (48.6% in the control group and 55.1% in the intervention group) [4]. Moreover, most of the smokers in China consumed 11–20 cigarettes per day [13, 18], while in Indonesia the average of cigarettes smoked per day was 15 [21]. Studies in China showed a different variation of time to the first cigarette after waking up on a day. A study by Elton-Marshall [18] found that they smoked their first cigarette in less than 5 min after waking up while another study by Fong [22] found that they started in between 5 to 30 min. Similarly, in Malaysia, Elton-Marshall [18] found that the smokers smoked their first cigarette in 6 to 30 min after waking up, while Fathelarahman [4] found that they started after 60 min they woke up in the morning both in control and intervention groups. The study in Indonesia shows that most of the adult smokers started their first smoke after 60 min as they woke up in the morning [21]. Further demographic characteristics and smoking behaviours measured in the reviewed studies can be seen in Table 3.

**Table 3:** Demographic Characteristics and Smoking Behaviour of Respondents

First Author	Gender of Respondents	Age range (years)/ mean age (years)	Area of Study Location	Education range/level	Occupation	Income range	Smoking behaviour			
							Smoking Frequency	Types of Cigarette	Highest % of time to first cig	Highest % of cig per day/average
Fathelrahman et al.	Male only	≥ 18	–	(not significant)	(not significant)	(not significant)	At least weekly smokers	–	> 60 min (45.7% in control group & 39.7% in intervention group)	11–20 (48.6% in control group & 55.1% in intervention group)
Fong et al.	Male and Female	13–17; and ≥ 18	Urban	–	–	< 3000 yuan; 3000–6999 yuan; > = 7000 yuan; no answer	Adult: smokers; non-smoker Youth: never smoker, former smoker, non-daily smoker; daily smoker	–	5–30 min (35%)	–
Zaidi et al.	Male and Female	17	Urban	High school	–	–	Current smokers; non-smokers	Tobacco smoke and sisha smoke	–	–
Hawari et al.	Male and Female	17–26	–	College	Students	–	regular smokers; occasional smokers; and non-smokers	–	–	–
Yong et al.	Male and Female	18- ≥ 55	Rural and urban	≤ secondary ≥ secondary	–	Low, moderate, high; not stated	Nondaily smoker Daily smoker	RYO and FM Cigarettes	–	Malaysia: 0–10 (55.9%); Thailand:11–20 (47%)
Behera et al.	–	18–25; and 26- ≥ 45	Rural and urban	Illiterate to graduate or above	varied	–	Never consume; Smoking only; both smoking and Chewing	–	–	–
Tugrul, Tugba Orten	Male and Female	19–25	Urban	University	Students	–	non-smokers; 1st stage, 2nd stage, 3rd stage smokers	–	–	–
Awaisu et al.	Male and Female	18- ≥ 41	Urban	Middle school -graduate; and other	Governmental sector; private sector; unemployed; and others	–	never smoker; current smoker; ex-smoker	–	–	–
Wu et al.	Male and Female	< 25; and 25- ≥ 55	Urban	Elementary to graduate or above	varied	≤10,000 RMB; 10.000- ≥ 50,000 RMB	non-smokers daily smokers occasional smokers	–	–	–
Sychareun et al.	Male and Female	15–55	Urban; Semi urban; Rural	Illiterate to Master/PhD	varied	–	Non-smoker Ex-smokers Daily smokers Occasional smokers	–	–	–
Elton-Marshall et al.	Male and Female	18–24; and 25- ≥ 55	–	Low to high; and not stated	–	Low, moderate, high; not stated	Daily smokers weekly smokers	–	Malaysia: 6–30 min (25.4%) China: ≤ 5 min (27.5%)	Malaysia: 0–10 (47.2%) China: 11–20 (48.3%)
Auemaneekul et al.	Male and Female	15–24	–	School and Collage	–	–	never smoker; current smoker; ex-smokers	–		
Dien et al.	Male and Female	15–18; and ≥19	Rural and urban	High school (youth) and low to high (adult)	–	≤minimum wage; ≥minimum wage	Youth: Smokers and non-smokers Adult: smokers	–	≥60 min (39%)	15
Mutti et al.	Male and Female	16–18; and ≥19	–	Low to graduate (youth) and illiterate to graduate or above (adult)	–	Low, moderate, high; not stated	Youth: everyday user; non-daily-user; non-user susceptible; non-user non-susceptible; Adult: everyday user; non-daily-user	Smoked tobacco, smokeless tobacco, mixed (smoked and smokeless)	–	–

Empty cells indicate the component was not identified

### Reaction to pictorial warnings

Generally, respondent’s reactions to pictorial warnings in the reviewed articles can be classified into three categories: salience (reading, looking at or noticing the warning), emotional reaction, and cognitive reaction as shown in Table 4. A study in Jordan found that all proposed pictorial warnings in the study had greater proportions of perception of salience among both non-smokers and smokers compared with the currently-implemented pictorial warning which was less shocking and has a smaller size [23]. In Malaysia, following the implementation of new graphic health warning, the study by Fathelrahman, et al. [4] found that the association between the pictorial warnings and the increase in avoiding looking at or thinking about the label was significant in both control and intervention groups (ps = 0.003 and < 0.001 respectively). However, the increase in the intervention group after being exposed to the pictorial warning was much higher than that in the control group after being exposed to the text-only warning (40.6% and 21.4% increases).

**Table 4:** Respondents’ Reaction to Measured Warnings

Study	Country	Smoking status	Age groups	Salience	*p* value	Emotional Reaction	*p* value	Cognitive Reaction	*p* value
Hawari et al.	Jordan	Nonsmokers	Young adult	Perceptions of salience between the picture of a child covering mouth vs current pictorial warnings	< 0.001	Fear elicitation between the picture of a coffin vs current pictorial warnings	< 0.001	Gaining of information between the picture of a child using inhaler vs current pictorial warnings	< 0.001
Hawari et al.	Jordan	Smokers	Young adult	Perceptions of salience between the picture of a child covering mouth vs current pictorial warnings	0.004	Fear elicitation between the picture of a coffin vs current pictorial warnings	< 0.001	Gaining of information between the picture of a child using inhaler vs current pictorial warnings	0.05
Tugrul, T.O.	Turkey	Nonsmokers	Youth	–		Fear	< 0.001	–	–
Tugrul, T.O.	Turkey	1st stage smokers	Youth	–		Fear	< 0.001	–	–
Tugrul, T.O.	Turkey	2nd stage smokers	Youth	–		Fear	< 0.001	–	–
Tugrul, T.O.	Turkey	3rd stage smokers	Youth	–		Fear	0.227	–	–
Tugrul, T.O.	Turkey	Nonsmokers	Youth	–		Disgust	0.027	–	–
Tugrul, T.O.	Turkey	1st stage smokers	Youth	–		Disgust	< 0.001	–	–
Tugrul, T.O.	Turkey	2nd stage smokers	Youth	–		Disgust	0.033	–	–
Tugrul, T.O.	Turkey	3rd stage smokers	Youth	–		Disgust	< 0.001	–	–
Fathelarahman et al.	Malaysia	Smokers	Adult	Reading or looking closely after exposure to PHW	0.607	–	–	Think of harm after exposure to PHW	0.004
Fathelarahman et al.	Malaysia	Smokers	Adult	Avoid looking after exposure to PHW	< 0.001	–	–	The change of knowledge after exposure to PHW	< 0.001
Yong et al	Thailand	Smokers	Adult	Notice after exposure to new PHW	< 0.001	–	–	Think of health risk after exposure to new PHW	< 0.001
Yong et al	Thailand	Smokers	Adult	Read after exposure to new PHW	< 0.001	–	–	–	–
Yong et al	Thailand	Smokers	Adult	Avoid looking after exposure to new PHW	< 0.001	–	–	–	–
Yong et al	Malaysia	Smokers	Adult	Notice after exposure to new text-only warning	not sig.	–	–	Think of health risk after exposure to new PHW	not sig.
Yong et al	Malaysia	Smokers	Adult	Read after exposure to new text-only warning	not sig.	–	–	–	–
Yong et al	Malaysia	Smokers	Adult	Avoid looking after exposure to new text-only warning	not sig.	–	–	–	–
Yong et al	Thailand vs. Malaysia	Smokers	Adult	Avoid looking after exposure to new label	< 0.01	–	–	–	–
Yong et al	Thailand vs Malaysia	Smokers	Adult	Notice after exposure to new label	< 0.001	–	–	–	–
Yong et al	Thailand vs Malaysia	Smokers	Adult	Read after exposure to new label	< 0.05	–	–	–	
Behera et al.	India	Smokers	Adult	Notice or not after exposure to new PHW	> 0.05	–	–	–	–
Behera et al.	India	Nonsmokers	Adult	Notice or not after exposure to new PHW	> 0.05	–	–	–	–
Awaisu et al.	Qatar	Non-smokers vs smokers	Adult	–		Fear	0.233	Gaining of more information compared with text-only warning	0.03
Elton-Marshall et al.	China vs. Malaysia	Smokers	Adult	Noticing after changes of warnings	0.02	–	–	Thinking about health risks after changes of warnings	0.13
Elton-Marshall et al.	China vs. Malaysia	Smokers	Adult	Reading/looking closely after changes of warnings	0.04	–	–	–	–
Elton-Marshall et al,	China vs. Malaysia	Smokers	Adult	Avoid looking/thinking after the changes of warnings	0.02	–	–	–	–

Empty cells indicate the component was not identified

In line with that, in Malaysia, Elton-Marshall et al. [18] also found significant in the change in noticing, reading and avoiding looking at/thinking of the label following the new pictorial warning compared with China text-only warning (ps = 0.02, 0.04, 0.02 respectively). Prior to the new pictorial warning, Malaysia had implemented text-only warning until January 2009 which was not changed over a period of time [4, 13]. Meanwhile, here was no significant change in noticing, reading and avoiding looking at the label over the period of the implementation of text-only warning in Malaysia [13]. On the other hand, the study found significant in avoiding looking at the label following the new graphic health warning in Thailand compared with their Malaysian counterparts (*p* value < 0.01). The same pattern also applied to the change in noticing and reading the label in the comparison between Thai pictorial warning and Malaysian text-only warning (ps < 0.001 and < 0.05) [13]. On the other hand, In India, noticing the pictorial warning on tobacco product was not associated with smoking behaviour [24].

Several studies also measured the emotional effects of pictorial warning vs. text-only warning or new pictorial warning vs. the old ones. The study conducted in young Jordanian adult found that the picture of a coffin elicited more fear in both smokers and non-smokers compared with the current pictorial warning which showed a diseased lung (*p* value < 0.001) [23]. Similarly, the study in Turkish youth showed that fear and disgust were evoked by the exposure to pictorial warning label among smokers and non-smokers [25]. The fear elicitation effect was also shown among Qatari Adult after being exposed to the pictorial warning [20].

In the reviewed studies, the cognitive effects such as the gain of knowledge or information and thinking of harm of smoking had been found significantly associated with the exposure to the pictorial warnings. The study in young Jordanian adult yielded a significantly greater proportion of gained information after being exposed to a child using inhaler compared with the current-lung-diseased-warning in both smokers and non-smokers (ps = 0.05 and < 0.001 respectively) [23]. Similarly, Malaysian-adult smokers thought of harm (*p* value = 0.004) and showed the change of knowledge (*p* value = < 0.001) after being exposed to the pictorial warning [16]. In line with that, a study in Qatari adult yielded gained information after the exposure to pictorial warning vs. exposure to text only warning (0.03). However, the thought of health risk was not found significantly associated with the exposure of pictorial warning nor significantly different between the change of text-only warning to the pictorial warning in Malaysia and the change of the old text-only warning to the new one in China (*p* value = 0.13) [13, 18, 23].

### Perceived effectiveness of PHW as a deterrent to smoking intention

In the reviewed articles, the perceived effectiveness of pictorial warning as a deterrent of smoking intention was mostly assessed in both smokers and non-smokers. In non-smokers, the perceived effects of pictorial warnings were evaluated in deterring smoking initiation among youth. Meanwhile, in smokers, the perceived effects of pictorial warnings were examined in restraining them to start smoking. Aumaneekul et al. [26] compared pictorial warning and the plain packaging which resulted in greater intention not to smoke in non-smokers and ex-smokers after exposure to plain packaging compared with the current smokers (*p* value < 0.05). Similarly, in Indonesia, the level of confidence to avoid smoking in the future in youth non-smokers was found significantly different between before and after exposure to pictorial warning (*p* value < 0.05) [21]. Moreover, a study in India and Bangladesh also showed that the perceived effectiveness of graphic warning was rated higher than the symbolic and testimonial warnings (*p* value < 0.01) [19]. In contrary, the study in Jordan did not find any significant difference between the proposed pictorial warnings compared with the current warning [21]. In line with that, Tugrul et al. [25] did not find any differences between female and male respondents in perceiving the effectiveness of pictorial warning in motivating not to smoke among non-smokers and among those who ever consider smoking (ps = 0.561 and 0.424 respectively).

### Perceived effectiveness of PHW as a stimulant of smoking cessation

The study by Hawari et al. [23] yielded a significant difference between female and male smokers in perceiving the effectiveness of pictorial warning in motivating not to start smoking and to quit smoking (ps = 0.019 and 0.002, respectively). The perception was significantly indicated by fear rather than disgust [23]. In Indonesia, the study by Dien et al. [21] revealed that the differences of the level of confidence to stop smoking in the future between before and after the exposure to pictorial warnings were found significant with *p* value < 0.001 in both youth and adult smokers. Similarly, Mutti et al. [19] found that the perceived effectiveness of graphic warning on smoking cessation compared with symbolic and testimonial warning was significantly different (ps = < 0.001).

In Malaysia, fathelarahman et al. [4] examine the perceived effectiveness of pictorial warnings in three different variables: think to quit smoking, no interest in quitting smoking; and interested in quitting smoking within the next month. All of the three variables were found significantly different between before and after exposure to pictorial warnings [4]. Fong et al. compared the old Chinese text-only warning with the new one and with several different types of warnings from different countries. The study found that pictorial warnings were rated higher in motivating to quit smoking compared to text-only warning (*p* value < 0.001). Similarly, Yong et al. [13] found that there were significant differences between the likely to quit smoking in wave 1 and wave 3 (after the implementation of new warning) with *p* values < 0.001 in Thailand and < 0.05 in Malaysia. Moreover, the study yielded the significant differences between the avoidance of cigarette in wave 1 and wave 3 with the *p* value < 0.001 in Thailand and Malaysia.

Awaisu et al. [26] compared non-smokers and smokers in perceiving pictorial warnings to alter smoking cessation behaviours, and the result found significantly different (*p* value < 0.001). Additionally, when comparing abstract and real pictures in influencing the intention to quit smoking, Wu, et al. [15] found a significant difference between those types (*p* value = 0.025). Moreover, the authors found that there was a significant difference between a picture with less graphic and that with more graphic (*p* value = 0.001). Similarly, Elton-Marshall, et al. [18] found that there was a difference after the changes of health warnings on cigarette packs (*p* value < 0.001) in China compared to that in Malaysia. The perceived effectiveness of pictorial health warnings measured by the studies can be seen in Table 5.

**Table 5:** Perceived Effects of Measured Warnings on Smoking Intention and Smoking Behaviour

Study	Country	Smoking status	Age groups	Perceived Effects on Smoking Intention	*p* value	Perceived Effects on Smoking-Behaviour	*p* value
Zaidi et al.	Pakistan	Smokers vs. non-smokers	Youth	picture of oral cavity cancer as deterrents from smoking compared with text-only warning	< 0.001	–	
Zaidi et al.	Pakistan	Smokers vs. non-smokers	Youth	picture of cancerous lungs as deterrents from smoking compared with text-only warning	< 0.001	–	
Hawari et al.	Jordan	Non-smokers	Young adult	Motivation not to initiate smoking	not. Sig	Motivation to quit smoking between the picture of a child using inhaler vs current pictorial warnings	0.003
Tugrul, T.O.	Turkey	Non-smokers	Youth	effectiveness in motivating not to consider smoking in female vs male	0.561	–	
Tugrul, T.O.	Turkey	1st stage smokers	Youth	effectiveness in motivating not to try smoking in female vs male	0.424	–	
Tugrul, T.O.	Turkey	2nd stage smokers	Youth	–		effectiveness in motivating not to start smoking in female vs male	0.019
Tugrul, T.O.	Turkey	3rd stage smokers	Youth	–		effectiveness in motivating to quit smoking in female vs male	0.002
Auemaneekul et al.	Thailand	Nonsmokers vs. current smokers	Youth	Intention not to smoke after exposure to plain packaging	< 0.05	–	
Auemaneekul et al.	Thailand	ex-smokers vs. current smokers	Youth	Intention not to smoke after exposure to plain packaging	< 0.05	–	
Dien et al.	Indonesia	Nonsmokers	Youth	Level of confidence to avoid smoking in the future (before vs after exposure to pictorial warnings	< 0.05	–	
Dien et al.	Indonesia	Smokers	Youth	–		Level of confidence to stop smoking in the future (before vs after exposure to pictorial warnings	< 0.001
Dien et al.	Indonesia	Smokers	Adult	–		Level of confidence to stop smoking in the future (before vs after exposure to pictorial warnings	< 0.001
Mutti et al.	India & Bangladesh		Youth	Perceived effectiveness of graphic warning on smoking initiation compared with symbolic warning	< 0.001	–	
Mutti et al.	India & Bangladesh		Youth	Perceived effectiveness of graphic warning on smoking initiation compared with testimonial warning	< 0.001	–	
Mutti et al.	India & Bangladesh		Adult	–		Perceived effectiveness of graphic warning on smoking cessation compared with symbolic warning	< 0.001
Mutti et al.	India & Bangladesh		Adult	–		Perceived effectiveness of graphic warning on smoking cessation compared with testimonial warning	< 0.001
Fathelarahman et al.	Malaysia		Adult	–		Think to quit before vs. after exposure to PHW	0.017
Fathelarahman et al.	Malaysia		Adult	–		No interest in quitting before vs. after exposure to PHW	0.003
Fathelarahman et al.	Malaysia		Adult	–		Interested within the next month before vs. after exposure to PHW	0.003
Fong et al.	China		Adult	–		Motivation to quit smoking between non-Chinese pictorial warnings and text-only warnings	< 0.0001
Fong et al.	China		Adult	–		Motivation to quit smoking between non-Chinese text-only warnings and Chinese text-only warning	< 0.0001
Yong et al.	Thailand	Smokers	Adult	–		Likely to quit smoking after exposure to new PHW	< 0.001
Yong et al.	Malaysia	Smokers	Adult	–		Forgoing/avoiding cigarettes after exposure to new PHW	< 0.001
Yong et al.	Thailand	Smokers	Adult	–		Likely to quit smoking after exposure to new text-only warning	< 0.05
Yong et al.	Malaysia	Smokers	Adult	–		Forgoing/avoiding cigarettes after exposure to new text-only warning	< 0.001
Awaisu et al.	Qatar	Non-smokers vs. smokers	Adult	–		Altering smoking cessation behaviours	< 0.001
Wu et al.	China	Smokers	Adult	–		Intention to quit smoking from abstract vs real pictures	0.025
Wu et al.	China	Smokers	Adult	–		Intention to quit smoking from the picture of adult vs the picture of child	0.002
Wu et al.	China	Smokers	Adult	–		Intention to quit smoking from the picture of male vs the picture of female	0.033
Wu et al.	China	smokers	Adult	–		Intention to quit smoking from foreign vs domestic pictures	1
Wu et al.	China	Smokers	Adult	–		Intention to quit smoking from less graphic vs more graphic pictures	0.001
Sychareun et al.	Lao PDR	Non-smokers vs. smokers	Adult	–		Encourage to quit smoking compared with text-only warning	0.37
Elton-Marshall et al.	China vs. Malaysia	Smokers	Adult	–		Thinking about quitting before vs after changes of warnings	< 0.001
Elton-Marshall et al.	China vs. Malaysia	Smokers	Adult	–		forgoing cigarette at least once before vs after changes of warnings	< 0.001

Empty cells indicate the component was not identified

## Discussion

In this study, we explored original research findings on perceptions of the effectiveness of pictorial health warnings on cigarette packs in Asian countries with varying methods, study populations and components measured. Using a literature review, we analyse three main variables: people’s reactions to pictorial health warnings, perceived effectiveness of pictorial health warnings in deterring smoking initiation, and perceived effectiveness of pictorial health warnings in stimulating smoking cessation. This study is useful to evaluate the implementation of pictorial health warnings on cigarette packs in Asia.

This study found that there are limited adequate studies evaluating the effectiveness of Pictorial health warnings in Asian countries that were published online, meaning that there might be more studies on Pictorial health warnings that have not been published yet or were published in other languages besides English and Indonesian. However, the existing articles in this study could describe effectiveness of pictorial health warnings in Asian countries. Our finding is consistent with a report by The Union [27] revealing that Asian countries have made significant progress in implementing and strengthening pictorial health warnings. In line with that, our reviewed studies showed that several countries including China, Jordan, and Turkey proposed new pictorial warnings indicating those countries were strengthening pictorial health warnings on cigarette packs by updating the pictures. Furthermore, Thailand, which implemented pictorial warning labels on 50% of front and back of cigarette packs in 2005, increased the size of the warning up to 85% covered on both sides. It shows that some Asian countries exerted to implement a more effective pictorial health warning to overcome the impacts of smoking behaviour [27].

Another report revealed that almost all countries in southeast Asia have carried out an efficacy testing of their pictorial health warnings [28]. Similarly, our findings also showed that several studies had been conducted to evaluate the effectiveness of pictorial health warnings on smoking intention and smoking behaviour as well as people’s reactions to them. Our study found that new or larger pictorial warnings could increase the salience, cause more fear, and gain more information and knowledge about the health risk of smoking behaviour. Those effects were not only occurred among smokers but also non-smokers. A study in the United States showed that the graphic warning increased perceived harms [29]. A previous study also suggested that the characteristics of a warning might influence the extent to which the warning will be noticed and recalled, which later generate reactions [30]. A regular introduction of a new message or warning was known to be able to maintain or even increase warning salience [31]. New design labels with efficacy and threat messages can play a role in efficacy beliefs by affecting held beliefs salient, especially among low SES populations [32].

Furthermore, a study on individual-level psychological outcomes as a result of exposure to pictorial health warnings showed that the warnings might change the smoking-related intentions and behaviours [33]. Previous studies also revealed that graphic warning labels are obviously more effective than text-only labels in promoting changes in attitudes, beliefs, knowledge, intentions to quit as well as quit attempts [11, 34–36]. It is in line with our finding which shows that pictorial health warnings in Asian countries generated different effects on smoking behaviour when compared to text-only warnings. However, there are limited studies on the association between the increase in warning size and quit rate in Asia. As a result, the real efficacy, besides the perceived efficacy, of pictorial health warnings in Asia cannot be actually measured.

## Conclusion

Asian countries have significantly made prominent progress in implementing and strengthening Pictorial health warnings. The reviewed studies show the measurements of the perceived effectiveness of pictorial health warnings had been conducted in several countries. When comparing pictorial warnings and text-only warnings, all studies suggested that pictorial warnings are more effective in changing knowledge, attitude, salient, smoking intention, and quit intention. Moreover, the reviewed studies also revealed that larger and new pictorial warnings are required to be introduced regularly to maintain behaviour salience. However, the relationship between pictorial health warnings and quit rate needs to be measured in future studies, as well as the effective period to change or refresh pictorial health warnings.

## Abbreviations

FCTC: Framework Convention on Tobacco Control
PHWs: Pictorial Health Warnings
PRISMA: Preferred Reporting Items for Systematic Reviews and Meta-Analysis
SES: Socio-economic Status
WHO: World Health Organization
